# Association between Hemoglobin Concentration and the Progression or Development of Albuminuria in Diabetic Kidney Disease

**DOI:** 10.1371/journal.pone.0129192

**Published:** 2015-05-29

**Authors:** Hiroshi Okada, Goji Hasegawa, Muhei Tanaka, Takafumi Osaka, Yayoi Shiotsu, Hiromichi Narumiya, Mamoru Inoue, Koji Nakano, Naoto Nakamura, Michiaki Fukui

**Affiliations:** 1 Department of Metabolism, Nephrology and Immunology, Japanese Red Cross Kyoto Daini Hospital, 355-5 Haruobi-cho, Kamigyo-ku, Kyoto, 602-8026, Japan; 2 Department of Endocrinology and Metabolism, Kyoto Prefectural University of Medicine, Graduate School of Medical Science, Kyoto, Japan; 3 Department of Endocrinology and Metabolism, Kyoto Yamashiro General Medical Center, Kyoto, Japan; Mayo Clinic Arizona, UNITED STATES

## Abstract

**Aims:**

Anemia, which might contribute to pathogenesis of kidney dysfunction, is a common finding in patients with type 2 diabetes. The aim of this study was to investigate if hemoglobin concentration is associated with the degree of change in urinary albumin-creatinine ratio or the development of albuminuria in patients with type 2 diabetes.

**Methods:**

We measured hemoglobin concentration in 470 (296 men and 174 women) consecutive type 2 diabetic patients without albuminuria. We performed a follow-up study to assess the progression or development of albuminuria, the interval of which was 3.0 years. Then we evaluated relationships between hemoglobin concentration and albuminuria, using multivariate linear regression analyses and logistic regression analyses.

**Results:**

Eighty four patients developed albuminuria during follow-up duration. In multivariate analyses, hemoglobin concentration was negatively associated with a change in urinary albumin-creatinine ratio in men (*ß *= -0.259, *P *= 0.0002) and women (*ß *= -0.194, *P *= 0.030). Moreover, multivariate adjusted odds ratio associated with 1 g/L in hemoglobin for the development of albuminuria was 0.93 (95% confidence interval; 0.89-0.96) in men and 0.94 (95% confidence interval; 0.88-0.99) in women, respectively. And, multivariate analyses revealed that adjusted odds ratios for the development of albuminuria were 4.78 (95% confidence interval; 1.65-13.91) in men and 4.62 (95% confidence interval; 1.34-16.68) in women with anemia (hemoglobin < 130 g/L for men and < 120 g/L for women), which were higher than those without anemia.

**Conclusions:**

Low hemoglobin concentration could be a predictor for the progression and development of albuminuria in patients with type 2 diabetes.

## Introduction

Diabetic kidney disease is a common complication and the leading cause of end-stage renal failure in patients with type 2 diabetes.[1, 2] Previous studies have reported that albuminuria, the earliest manifestation of diabetic kidney disease, is a marker of increased cardiovascular mortality and the progression of cardiovascular disease.[3, 4] Therefore, the finding of albuminuria is an indication for screening for possible vascular disease and aggressive intervention to reduce all cardiovascular risk factors.[5]

On the other hand, anemia is the common finding in patients with type 2 diabetes.[6, 7] Thomas et al. [8] have suggested that the prevalence of the development of anemia in diabetic patients with albuminuria was higher than those without albuminuria in cohort study. Hemoglobin concentration is associated with the risk of cardiovascular disease and mortality in patients with or without diabetes.[9–11] In addition, some studies have demonstrated that low hemoglobin concentration might be associated with an increased risk of progression of kidney disease in patients with type2 diabetes, including patients with or without albuminuria.[12–15] One study has reported that low hemoglobin concentration is a predictor for decline in glomerular filtration rate in type 2 diabetic patients without albuminuria.[16] And, we have previously revealed negative correlation between hemoglobin concentration and urinary albumin-creatinine ratio (UACR) in men with type 2 diabetes in a cross-sectional study.[17] However, it is not unclear whether low hemoglobin concentration contributes to the progression or development of albuminuria in patients with type 2 diabetes. Therefore, we evaluated the relationship between hemoglobin concentration and the degree of change in UACR or the development of albuminuria in patients with type 2 diabetes.

## Methods

### Patients and study design

We performed a retrospective cohort study with 1188 patients with type 2 diabetes who had regularly attended the diabetes outpatient clinic at Japanese Red Cross Kyoto Daini Hospital, Kyoto Prefectural University of Medicine and Kyoto Yamashiro General Medical Center from April, 2006 to June, 2014. Type 2 diabetes was diagnosed according to the Report of the Expert Committee on the Diagnosis and Classification of Diabetes Mellitus.[18] Body mass index (BMI) was calculated as weight in kilograms divided by height in meters squared. Patients were classified as never smokers, previous smokers, or current smokers according to a self-administered questionnaire. Patients with albuminuria were defined as UACR equal to or more than 30 mg/g creatinine.[5] Out of 1188 patients with type 2 diabetes 718 patients were excluded from this study because of the presence of albuminuria at baseline (n = 394), no measurement of UACR at the end of the follow-up interval (n = 137), advanced renal dysfunction, defined as serum creatinine equal to or more than 2.0 mg/dL (n = 36), malignancy (n = 21), collagen disease (n = 4), hematologic disease (n = 7), the use of anemia treatment such as ferric citrate (n = 12) or the development of major cardiovascular event during a follow-up (n = 11). Moreover, we excluded patients (n = 96) who were newly prescribed rennin-angiotensin system (RAS) inhibitors such as angiotensin-converting enzyme inhibitors, angiotensin II receptor blockers and direct rennin inhibitors for the first time during follow-up period of this study because RAS inhibitors could delay the development of albuminuria in patients with diabetes.[5] We then evaluated relationships between hemoglobin concentration and the degree of change in UACR and calculated the odd ratio for development of albuminuria in patients with type 2 diabetes. This study was performed according to ethics statement by Japanese ministry of health, labour and welfare, and was conducted in accordance with Declaration of Helsinki. Japanese ministry of health, labour and welfare suggests that if data is anonymously retrieved from database, written consent and recording participant consent are not necessarily needed in clinical research and information disclosure about study should be performed instead of obtaining written consent and recording participant consent. We performed information disclosure to participants based on Japanese ministry of health, labour and welfare, and participants provided verbal informed consent to participate in this study including aim or methods of this study and handling of personal information. This study and consent procedure were approval by Ethics Committee of Japanese Red Cross Kyoto Daini Hospital.

### Data collection

All data had been retrieved from a database. Overnight fasting blood samples were taken in the morning at baseline. Blood and urine examinations including hemoglobin concentration had been done systematically at periodic visit in all patients. Anemia was defined as hemoglobin concentration less than 130 g/L in men and less than 120 g/L in women according to definition by the World Health Organization.[19] Glomerular filtration rate was estimated using the equation of Japanese Society Nephrology: estimated glomerular filtration rate = 194 × Cre^-1.094^ × age^-0.287^ (ml/min/1.73m^2^).[20] Serum total cholesterol and triglyceride concentrations were assessed using standard enzymatic methods. Hemoglobin A1c was assayed using high-performance liquid chromatography and expressed with the unit defined by the National Glycohemoglobin Standardization Program. Urinary albumin and creatinine concentration were determined in an early morning spot urine. UACR was measured with an immunoturbidimetric assay. As a follow-up study, we collected urine samples for calculation of UACR after 3 years. A mean value for UACR both at baseline and the end of the follow-up interval was determined from 3 urine collections. Change in UACR was calculated as follows; change in UACR = UACR at the end of the follow-up interval—UACR at baseline. The development of albuminuria was defined as UACR equal to or more than 30 mg/g Cr.[5]

### Statistical analysis

Means and frequencies or percentages of potential confounding variables were calculated. Because of the difference in hemoglobin concentration between sexes, the participants were separated to men and women. The differences of general characteristics at baseline and those according to the development of albuminuria at the end of follow-up were assessed by the chi-square test for categorical variables and by the unpaired Student t-test or Mann-Whitney’s U test for continuous variables, respectively. Because triglycerides showed skewed distributions, log transformation was carried out before performing correlation and regression analysis. The relationships between hemoglobin concentration and change in UACR as well as the relationships between hemoglobin concentration and other variables were examined by Pearson’s correlation analyses. To examine the effects of various factors on change in UACR or the development of albuminuria defined as UACR equal to or more than 30 mg/g Cr, the following factors which was statistically significant in univariate analysis and those known to be related factors for the development of albuminuria were considered simultaneously as independent variables for multivariate linear regression analysis: Model 1; age, duration of diabetes, BMI, systolic blood pressure (SBP), hemoglobin and hemoglobin A1c, Model 2; all variables in Model 1 plus total cholesterol, logarithm of triglycerides, uric acid, creatinine, smoking status, the use of RAS inhibitors and the use of statin, or for multivariate logistic regression analysis: Model 1; age, duration of diabetes, SBP, hemoglobin, hemoglobin A1c, UACR at baseline and the use of RAS inhibitors, Model 2; all variables in Model 1 plus BMI, total cholesterol, logarithm of triglycerides, uric acid, creatinine, smoking status and the use of statin. Receiver-operating characteristic (ROC) curves were constructed to assess the ability to identify the development of albuminuria. The areas under the ROC curves were compared by a nonparametric approach.

All continuous variables are presented as the mean ± SD, absolute number or percentages. A *P* value < 0.05 was considered statistically significant. The size, direction, and statistical significance of relationships were estimated by the hazard ratio with 95% confidence interval (CI). The statistical analyses were performed using the JMP version 10 software (SAS Institute Inc., Cary, North Carolina).

## Results

Characteristics of the 470 patients (296 men and 174 women) with type 2 diabetes enrolled in this study are shown in Table 1. Mean hemoglobin was 137.9 ± 13.8 g/L in overall. Mean hemoglobin was higher in men (143.1 ± 12.3 g/L) than women (129.1 ±.11.6 g/L; *P* < 0.0001). 84 patients (44 men and 40 women) developed albuminuria during the study period. We have analyzed the characteristics at baseline according to the patients with or without the development of albuminuria in men or women. Average SBP (*P* = 0.001) or UACR (*P* < 0.0001) was higher and hemoglobin was lower (*P* = 0.0003) at baseline in patients with the development of albuminuria than those without in men. Patients with the development of albuminuria more frequently used RAS inhibitor at baseline in men (*P* = 0.028). Age (*P* = 0.023), average SBP (*P* = 0.041), hemoglobin A1c (*P* < 0.0001), creatinine (*P* = 0.002) or UACR (*P* = 0.0002) was higher, duration of diabetes was longer (*P* = 0.008) and hemoglobin was lower (*P* < 0.0001) at baseline in patients with the development of albuminuria than those without in women.

**Table 1:** Characteristics of patients at baseline.

	Overall	Men	Women
n	470	296	174
Age (y)	64.2 (11.1)	63.5 (11.2)	65.5 (10.9)
Duration of diabetes (y)	12.2 (8.9)	12.1 (9.5)	12.3 (7.9)
Body mass index (kg/m^2^)	24.4 (4.3)	24.1 (4.1)	24.8 (4.6)
Systolic blood pressure (mmHg)	130.8 (17.4)	130.5 (16.8)	131.2 (18.4)
Diastolic blood pressure (mmHg)	74.4 (11.2)	75.5 (10.3)	72.9 (11.4)*
Hemoglobin (g/L)	137.9 (13.8)	143.1 (12.3)	129.1 (11.6)*
Anemia (%)	16.0	11.1	24.1*
Hemoglobin A1c (%)	7.2 (1.3)	7.2 (1.4)	7.2 (1.3)
Total cholesterol (mmol/L)	4.8 (0.8)	4.8 (0.8)	4.8 (0.8)
Triglycerides (mmol/L)	1.6 (1.0)	1.6 (1.1)	1.5 (0.9)
Uric acid (μmol/L)	309.3 (83.3)	327.1 (83.3)	279.6 (77.3)*
Creatinine (μmol/L)	67.2 (16.8)	73.4 (15.0)	56.6 (15.0)*
Estimated GFR (ml/min/1.73m^2^)	76.1 (19.3)	76.2 (19.3)	76.0 (19.2)
Smoking (%) (never/previous/current)	58.5/23.0/18.5	40.2/33.8/26.0	89.7/4.6/5.7*
UACR (mg/g creatinine) at baseline	12.1 (7.5)	12.0 (7.5)	12.4 (7.5)
UACR (mg/g creatinine) after 3 years	21.0 (33.0)	19.7 (32.9)	23.2 (33.2)
Development of albuminuria (%)	17.9	14.9	23.0*
History of cardiovascular disease (%)	16.2	17.6	13.8
Antidiabetic treatment (%)			
Diet only	8.5	8.4	8.6
Oral hypoglycemic agent	80.0	80.7	78.7
Insulin	29.5	22.0	35.6*
Hypertension (%)	63.4	61.3	66.7
Antihypertensive drugs (%)			
Calcium channel blockers	26.2	22.3	32.2*
Rennin-angiotensin system inhibitors	38.5	35.1	44.3*
Others	17.7	15.5	21.3
Statin (%)	51.1	44.4	62.1*

Data are expressed as mean (SD) or percent of patients. UACR, urinary albumin-creatinine ratio; GFR, glomerular filtration rate;

* *P* < 0.05 vs men

Table 2 showed the correlation between hemoglobin concentration and other variables. Hemoglobin concentration positively correlated with BMI, hemoglobin A1c, total cholesterol or logarithm of triglycerides, and negatively correlated with age, duration of diabetes, creatinine or UACR after 3 years in men. Hemoglobin concentration positively correlated with total cholesterol and negatively correlated with age, duration of diabetes, creatinine or UACR after 3 years in women.

**Table 2:** Correlation between hemoglobin and other variables at baseline.

	Men		Women	
	*r*	*P*	*r*	*P*
Age	-0.341	< 0.0001	-0.301	< 0.0001
Duration of diabetes	-0.242	< 0.0001	-0.254	0.0007
Body mass index	0.250	< 0.0001	0.066	0.388
Systolic blood pressure	0.050	0.396	-0.089	0.245
Hemoglobin A1c	0.231	< 0.0001	-0.029	0.706
Total cholesterol	0.156	0.007	0.187	0.014
Logarithm of triglycerides	0.181	0.002	0.009	0.903
Uric acid	-0.058	0.317	0.043	0.575
Creatinine	-0.143	0.014	-0.227	0.003
UACR at baseline	-0.020	0.756	-0.089	0.324
UACR after 3 years	-0.168	0.004	-0.216	0.005

UACR, urinary albumin-creatinine ratio

Simple and multivariate regression analyses on change in UACR are shown in Table 3. SBP or hemoglobin A1c positively correlated with change in UACR and hemoglobin negatively correlated with change in UACR in men. Duration of diabetes, BMI, SBP or hemoglobin A1c positively correlated with change in UACR and hemoglobin negatively correlated with change in UACR in women. Multivariate regression analyses demonstrated that SBP, hemoglobin A1c or hemoglobin was independently associated with change in UACR in men or women (Model 2).

**Table 3:** Simple correlation and multivariate regression analyses on change in urinary albumin-creatinine ratio.

	Crude		Model 1		Model 2	
Men	*r*	*P*	*ß*	*P*	*ß*	*P*
Age	0.070	0.267	0.012	0.862	0.014	0.842
Duration of diabetes	0.020	0.752	-0.034	0.540	0.004	0.949
Body mass index	0.030	0.633	0.024	0.722	0.037	0.611
Systolic blood pressure	0.164	0.009	0.148	0.019	0.133	0.039
Hemoglobin	-0.198	0.002	-0.254	0.0002	-0.259	0.0002
Hemoglobin A1c	0.151	0.016	0.176	0.007	0.202	0.003
Total cholesterol	-0.002	0.973	_	_	-0.004	0.954
Logarithm of triglycerides	0.031	0.624	_	_	0.001	0.989
Uric acid	0.066	0.298	_	_	0.085	0.196
Creatinine	0.054	0.395	_	_	0.019	0.780
Smoking status	_	_	_	_	0.010	0.874
RAS inhibitors	_	_	_	_	-0.012	0.859
Statin	_	_	_	_	-0.117	0.080
Women	*r*	*P*	*ß*	*P*	*ß*	*P*
Age	0.144	0.074	0.058	0.402	0.020	0.816
Duration of diabetes	0.269	0.0007	-0.040	0.545	0.128	0.135
Body mass index	0.228	0.005	0.083	0.211	0.233	0.004
Systolic blood pressure	0.304	0.0001	0.101	0.110	0.221	0.006
Hemoglobin	-0.276	0.0005	-0.230	0.0007	-0.194	0.030
Hemoglobin A1c	0.267	0.0008	0.134	0.035	0.175	0.040
Total cholesterol	-0.068	0.402	_	_	-0.025	0.774
Logarithm of triglycerides	-0.007	0.935	_	_	-0.008	0.925
Uric acid	0.077	0.345	_	_	0.034	0.715
Creatinine	0.138	0.089	_	_	0.024	0.793
Smoking status	_	_	_	_	-0.036	0.645
RAS inhibitors	_	_	_	_	-0.128	0.099
Statin	_	_	_	_	0.061	0.467

RAS, rennin-angiotensin system; Model 1 is adjusted for age, duration of diabetes, body mass index, systolic blood pressure, hemoglobin and hemoglobin A1c. Model 2 includes all variables in Model 1 plus total cholesterol, logarithm of triglycerides, uric acid, creatinine, smoking status, the use of rennin-angiotensin system inhibitors and the use of statin. Smoking status was defined as nonsmoker (= 0), previous smoker (= 1), or current smoker (= 2), and the use of rennin-angiotensin system inhibitors or statin was defined as without (= 0) or with (= 1).

The results of the unadjusted and adjusted logistic regression analyses are shown in Table 4. The unadjusted and adjusted logistic regression analysis revealed that hemoglobin concentration or the presence of anemia was associated with an increased odds of the development of albuminuria in men or women. Moreover, the unadjusted logistic regression analyses revealed that SBP (odds ratio; 1.03, 95% CI; 1.01–1.05), hemoglobin A1c (odds ratio; 1.30, 95% CI; 1.16–1.50) or UACR at baseline (odds ratio; 1.10, 95% CI; 1.06–1.16) was associated with an increased odds of the development of albuminuria in men and age (odds ratio; 1.04, 95% CI; 1.007–1.08), duration of diabetes (odds ratio; 1.06, 95% CI; 1.01–1.11), SBP (odds ratio; 1.02, 95% CI; 1.0005–1.04), hemoglobin A1c (odds ratio; 1.77, 95% CI; 1.36–2.36), creatinine (odds ratio; 24.59, 95% CI; 2.85–281.95) or UACR at baseline (odds ratio; 1.10, 95% CI; 1.05–1.17) was associated with an increased odds of the development of albuminuria in women. The areas under the ROC curves for SBP, hemoglobin, hemoglobin A1c and UACR at baseline regarding identification of the development of albuminuria were 0.63, 0.67, 0.60 and 0.72 in men. There were no significant differences between the area under the ROC curve for hemoglobin and those for other factors in men. The areas under the ROC curves for age, duration of diabetes, SBP, hemoglobin, hemoglobin A1c, creatinine and UACR at baseline regarding identification of the development of albuminuria were 0.60, 0.63, 0.61, 0.70, 0.70, 0.62 and 0.69 in women. The area under the ROC curve was significantly greater for hemoglobin than for age or creatinine (*P* = 0.017 or *P* = 0.032). And, the adjusted logistic regression analyses revealed that SBP (odds ratio; 1.02, 95% CI; 1.001–1.05) or UACR at baseline (odds ratio; 1.11, 95% CI; 1.05–1.17) was associated with an increased odds of the development of albuminuria in men and SBP (odds ratio; 1.04, 95% CI; 1.005–1.08), hemoglobin A1c (odds ratio; 2.01, 95% CI; 1.24–3.58) or UACR at baseline (odds ratio; 1.18, 95% CI; 1.08–1.31) was associated with an increased odds of the development of albuminuria in women, adjusted following factors; age, duration of diabetes, BMI, SBP, hemoglobin, hemoglobin A1c, total cholesterol, logarithm of triglycerides, uric acid, creatinine, smoking status, UACR at baseline, the use of RAS inhibitors and the use of statin.

**Table 4:** Unadjusted odds ratios and multivariate adjusted odd ratios for development of albuminuria.

Hemoglobin, per 1.0 g/L increase				
	Men	*P*	Women	*P*
Crude	0.95 (0.93–0.98)	0.0005	0.93 (0.89–0.96)	< 0.0001
Model 1	0.94 (0.90–0.97)	0.0001	0.93 (0.88–0.97)	0.002
Model 2	0.93 (0.89–0.96)	< 0.0001	0.94 (0.88–0.99)	0.013
Anemia, yes				
	Men	*P*	Women	*P*
Crude	4.13 (1.82–9.1)	0.0005	6.97 (3.22–15.51)	< 0.0001
Model 1	4.32 (1.60–11.62)	0.004	4.30 (1.54–12.62)	0.005
Model 2	4.78 (1.65–13.91)	0.004	4.62 (1.34–16.68)	0.013

Model 1 is adjusted for age, duration of diabetes, systolic blood pressure, hemoglobin, hemoglobin A1c, urinary albumin-creatinine ratio at baseline and the use of rennin-angiotensin system inhibitors. Model 2 includes all variables in Model 1 plus body mass index, total cholesterol, logarithm of triglycerides, uric acid, creatinine, smoking status, and the use of statin. Smoking status was defined as nonsmoker (= 0), previous smoker (= 1), or current smoker (= 2), and the use of rennin-angiotensin system inhibitors or statin was defined as without (= 0) or with (= 1).

## Discussion

The major finding of our study is that low hemoglobin concentration is a predictor for the progression or development of albuminuria in patients with type 2 diabetes after adjustment for the other risk factors. Hemoglobin concentration is lower in patients with diabetes than those without diabetes in general population.[21] Craig et al. [22] reported that diabetic patients even without albuminuria had an ongoing and decrease in hemoglobin concentration. Previous studies have reported that anemia is a predictor of further progression of advanced kidney disease in type 2 diabetic patients with or without albuminuria.[14, 16] Previously, we have revealed negative correlation between hemoglobin concentration and UACR in men with type 2 diabetes in a cross-sectional study. However, it was unclear if low hemoglobin concentration is a predictor for the progression or development of albuminuria. In this study, multivariate regression analyses have revealed the association between hemoglobin concentration and the progression or development of albuminuria in patients with type2 diabetes.

The erythropoietin dysregulation, caused by early damage to renal tubules, has been suggested as one of contributors to anemia in patients with diabetes.[23–27] Moreover, nutrient deficiencies, inflammation, concomitant autoimmune disease, drugs and hormonal changes might contribute to anemia in patients with diabetes.[28, 29] The mechanisms by which low hemoglobin concentration might contribute to the progression or development of albuminuria in patients with type 2 diabetes are unclear. There are some reports which could explain the association between low hemoglobin concentration and the progression or development of albuminuria. First, low hemoglobin concentration causes chronic renal hypoxia in tubulointerstitial area. Chronic renal hypoxia as well as oxidative stress or inflammation induces renal cell growth and extracellular matrix synthesis through activating several genes such as transforming growth factor-β1, osteopontin and hypoxia inducing factor-1 or inhibiting nitric oxide synthase.[30, 31] Therefore, chronic renal hypoxia is recognized to play an important role in tubulointerstitial damage.[32–34] Recently, it is appreciated that tubulointerstitial damage could play an important role in the pathogenesis of albuminuria.[34, 35] Next, anemia induces low renal blood flow thorough cardiac disorders. It was suggested that low hemoglobin concentration was closely associated with left ventricular hypertrophy or heart failure. Left ventricular hypertrophy has been reported to occur early in the course of diabetic kidney disease.[36, 37] Low renal blood flow might induce chronic hypoxia in tubulointerstitial areas.

There are several limitations in this study. Duration of hypertension could be also clinically significant variable in terms of diabetic kidney disease and physical exercise may affect UACR. And, there are potential contributors to anemia in patients with diabetes, such as undetected malignant disease, nutrient condition, chronic inflammation, autonomic neuropathy, reduced red cell survival or hypothyroidism.[29, 38] Unfortunately, however, we have no data about duration of hypertension, physical exercise or potential contributors to anemia. Moreover, low hemoglobin concentration was closely associated with left ventricular hypertrophy which has been reported to occur early in the course of diabetic kidney disease.[36, 37] It could be useful to add data on left ventricular hypertrophy and dysfunction in order to better investigate the relationship between hemoglobin concentration and the progression or development of albuminuria in patients with type 2 diabetes. Unfortunately, however, we have no data about left ventricular hypertrophy and dysfunction. Our study includes some patterns of RAS inhibitors at baseline, which could impact on the development of albuminuria. The study population consisted of Japanese men and women, therefore, it is uncertain whether these findings are generalized in other ethnic groups. In addition, the number of patients available for analysis was not large and the follow-up was rather short and the number of patients who developed albuminuria was small in this study. This study is retrospective study which can genuinely affect the result because patients who stay in hospital might be more severely diseased than those who go away for any reasons. Moreover, since this study is epidemiological, the relationship between hemoglobin concentration and the progression or development of albuminuria may not be causal in patients with type 2 diabetes. Anemia might simply be a marker of diabetic complications. Large prospective trials are needed to better assess the relationship between hemoglobin concentration and the progression or development of albuminuria in patients with type 2 diabetes.

The menstrual period is significantly associated with hemoglobin concentration in women. Therefore, it is important that how many premenopausal women were included in our study. Unfortunately, we had no exact data how many premenopausal women were included, but only 5.7% female subjects were under 50 years, who were assumed to be premenopausal. Multivariate regression analysis demonstrated that hemoglobin concentration was independently associated with change in UACR in women over 50 years (*ß* = -0.191, *P* = 0.033, Model 2). Adjusted logistic regression analyses demonstrated that hemoglobin concentration (odds ratio; 0.93, 95% CI; 0.88–0.98) or the presence of anemia (odds ratio; 5.17, 95% CI; 1.49–19.6) was associated with an increased odds of the development of albuminuria in women over 50 years (Model 2).

Hemoglobin could have an important role for the progression or development of albuminuria, however testing for hemoglobin concentration is not considered by National Institute for Health and Clinical Excellent guidance, nor by American Diabetes Association guidance.[39, 40] Then, we might have to test for hemoglobin concentration and patients with low hemoglobin concentration might require aggressive lifestyle modifications and medication to lower blood glucose and blood pressure for the prevention of diabetic kidney disease. To the best of our knowledge, this is the first study to investigate if hemoglobin concentration could be a predictor for the progression or development of albuminuria in patients with type 2 diabetes.

In conclusion, low hemoglobin concentration could be a novel predictor for the progression or development of albuminuria.
